# M. POWELL LAWTON AWARD LECTURE

**DOI:** 10.1093/geroni/igz038.1598

**Published:** 2019-11-08

**Authors:** Sharon Inouye

**Affiliations:** Harvard Medical School Aging Brain Center, Boston, Massachusetts, United States

## Abstract

The M. Powell Lawton Award is presented annually to an individual who has made outstanding contributions from applied research that has benefited older people and their care. The lecture will be given by the 2018 recipient, Carol Whitlatch, PhD, Benjamin Rose Institute on Aging. The session will also include the presentation of the 2019 Lawton Award. The 2019 Lawton Award recipient is Barbara Resnick, PhD, CRNP, FGSA, of the University of Maryland. Supported by the Polisher Research Institute of the Madlyn and Leonard Abramson Center for Jewish Life.

